# Low BCL7A expression predicts poor prognosis in ovarian cancer

**DOI:** 10.1186/s13048-019-0518-0

**Published:** 2019-05-10

**Authors:** Ziqian Sun, Liang Sun, Miao He, Ying Pang, Zhaoying Yang, Junrong Wang

**Affiliations:** 1 Department of Gynaecology and Obstetrics, China-Japan Union Hospital of Jilin University, 126 Xiantai Street, Changchun, 130033 People’s Republic of China; 2 Department of Breast Surgery, China-Japan Union Hospital of Jilin University, 126 Xiantai Street, Changchun, 130033 People’s Republic of China; 3 Department of Anesthesia, The Second Hospital of Jilin University, Changchun, 130022 People’s Republic of China

**Keywords:** Ovarian cancer, Prognosis, BCL7A, Data mining

## Abstract

**Background and objective:**

Ovarian cancer is a common gynaecological cancer with a poor prognosis that poses a serious threat to human life and health. It is essential to explore the possible prognostic biomarkers of ovarian cancer. As an important tumour suppressor gene, BCL7A actively participates in the growth of tumours. We aimed to study the prognostic role of BCL7A in ovarian cancer.

**Results:**

Through data mining of RNAseq data from the Cancer Genome Atlas database (TCGA), we explored the clinical relevance of BCL7A mRNA expression. As a result, we found that BCL7A is expressed at low levels in ovarian cancer tissues and is correlated with survival status. Survival analysis showed that, compared with those who had higher levels of BCL7A expression, patients with ovarian cancer and low levels of BCL7A generally had shorter overall/relapse-free survival times. Cox regression models showed that low BCL7A expression could be used as an independent prognostication factor for ovarian cancer patients.

**Conclusions:**

Low BCL7A expression is an independent risk factor for poor prognosis in ovarian cancer patients.

## Introduction

Ovarian cancer is a common gynaecological cancer; its mortality rate ranks first among gynaecological tumours and it poses a serious threat to human life and health [1, 2]. In most cases, ovarian cancer is usually detected in advanced stages, so even with advanced therapeutic strategies, such as targeted therapy, chemoradiotherapy, and combined chemotherapy, the overall 5-year survival rate of ovarian cancer is still poor [3–5]. Therefore, it is essential to explore the possible prognostic biomarkers of ovarian cancer.

As a key molecule of chromatin remodelling, BCL7A is involved in the process of carcinogenesis. BCL7A is expressed in the ovary, brain, lymph nodes and other tissues. The gene is located on chromosome 12q24.31, and 1 natural variant has been found so far. BCL7A plays an important role in human cancer. It has been found that Myc and IgH are directly involved in the three-way gene translocation of Burkitt lymphoma cell lines, which may contribute to the progression of Burkitt lymphoma [6, 7]. In addition, BCL7A was found to be highly methylated in patients with cutaneous T-cell lymphoma and was identified as a marker of a poor prognosis in early-stage cutaneous T-cell lymphoma patients [8]. However, the role of BCL7A in ovarian cancer remains unclear.

To further evaluate the clinical significance of BCL7A in the prognosis of ovarian cancer patients, we examined the differential expression of BCL7A mRNA in ovarian cancer by exploring the Cancer Genome Atlas database (TCGA). The chi-square test and Fisher’s exact test were used to evaluate the clinical correlation. Survival analysis and a Cox regression model were employed to identify the correlation between BCL7A and ovarian cancer patients’ survival rate.

## Results

### Data overview

BCL7A expression data and clinical features from the TCGA database, including age, lymphatic invasion, stage, histologic grade, subdivision, new type, longest dimension, sample type, vital status and BCL7A expression, are shown in Table 1.

**Table 1:** The baseline information of ovarian cancer patients from TCGA database

characteristics	Numbers.of.cases...
age	
< 55	113(36.69)
> =55	195(63.31)
subdivision	
NA	17(5.52)
Bilateral	212(68.83)
Left	37(12.01)
Right	42(13.64)
stage	
NA	2(0.65)
I	1(0.32)
II	22(7.14)
III	245(79.55)
IV	38(12.34)
longest dimension	
large	124(46.1)
small	145(53.9)
lymphatic invasion	
NA	180(58.44)
NO	44(14.29)
YES	84(27.27)
histologic grade	
NA	2(0.65)
G1	1(0.32)
G2	37(12.01)
G3	261(84.74)
G4	1(0.32)
GB	2(0.65)
GX	4(1.3)
sample type	
Primary Tumor	303(98.38)
Recurrent Tumor	5(1.62)
vital status	
DECEASED	184(59.74)
LIVING	124(40.26)
BCL7A	
high	154(50)
low	154(50)

### Differential expression of BCL7A in ovarian cancer

**Fig. 1:**
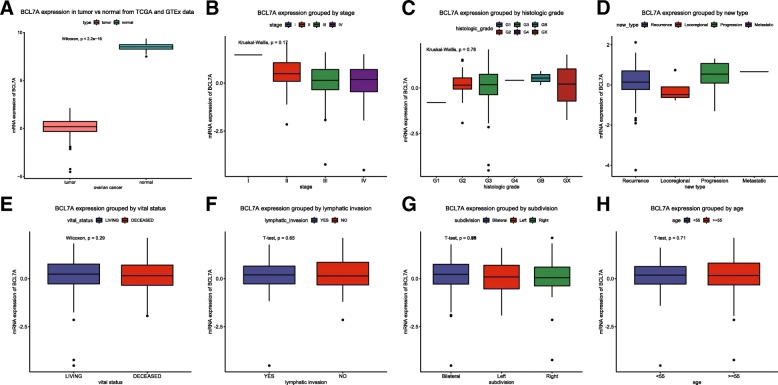
The different BCL7A expression levels shown in a boxplot. The differential expression of BCL7A in tumour vs normal tissues (**a**) and other groups according to stage (**b**), histologic grade (**c**), new type (**d**), vital status (**e**), lymphatic invasion (**f**), subdivision (**g**) and age (**h**)

Boxplots revealed that lower BCL7A expression was found in ovarian cancer tissues compared with normal ovarian tissues (Fig. 1). In addition, BCL7A expression gradually decreased with an increasing histologic grade.

We used the median of BCL7A expression as a cut-off to divide patients into a BCL7A high-expression group and a low-expression group, and there was no difference in the clinicopathological parameters between the two groups (Table 2).

**Table 2:** The correlation between BCL7A mRNA expression and clinical parameters

			BCL7A mRNA expression					
Parameter	Variable	N	high	%	low	%	χ2	Pvalue
age	< 55	113	57	(37.01)	56	(36.36)	0	1
	> = 55	195	97	(62.99)	98	(63.64)		
subdivision	Bilateral	212	111	(77.08)	101	(68.71)	3.0653	0.216
	Left	37	17	(11.81)	20	(13.61)		
	Right	42	16	(11.11)	26	(17.69)		
stage	I	1	1	(0.65)	0	(0)	2.8364	0.4175
	II	22	14	(9.15)	8	(5.23)		
	III	245	119	(77.78)	126	(82.35)		
	IV	38	19	(12.42)	19	(12.42)		
longest dimension	large	124	67	(49.26)	57	(42.86)	0.8682	0.3515
	small	145	69	(50.74)	76	(57.14)		
lymphatic invasion	NO	44	19	(31.15)	25	(37.31)	0.2995	0.5842
	YES	84	42	(68.85)	42	(62.69)		
histologic grade	G1	1	0	(0)	1	(0.65)	2.0309	0.8449
	G2	37	18	(11.76)	19	(12.42)		
	G3	261	131	(85.62)	130	(84.97)		
	G4	1	1	(0.65)	0	(0)		
	GB	2	1	(0.65)	1	(0.65)		
	GX	4	2	(1.31)	2	(1.31)		
sample type	Primary Tumor	303	152	(98.7)	151	(98.05)	0	1
	Recurrent Tumor	5	2	(1.3)	3	(1.95)		
vital status	DECEASED	184	89	(57.79)	95	(61.69)	0.3375	0.5613
	LIVING	124	65	(42.21)	59	(38.31)		

### A decrease in BCL7A expression is associated with a poor overall survival of ovarian cancer

**Fig. 2:**
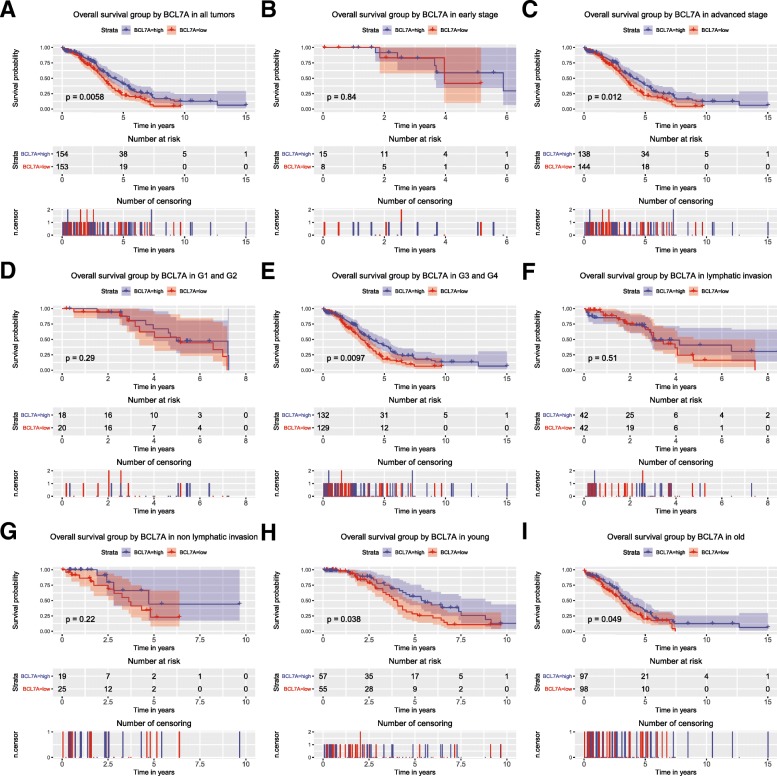
Survival analysis of BCL7A expression in terms of overall survival. Kaplan–Meier curves produced survival analysis (**a**) and subgroup analysis of early stage (**b**), advanced stage (**c**), histological grade G1/G2 (**d**) and G3/G4 (**e**), lymphatic invasion (**f**), non-lymphatic invasion (**g**), young patients (**h**) and older patients (**i**)

As shown in Fig. 2, patients with a shorter overall survival time had particularly low expression of BCL7A (*P* = 0.034), which was consistent with the results of subgroup analysis, especially for advanced stage (*P* = 0.012), G3 and G4 (*p* = 0.0097), young patients (*p* = 0.038) and older patients (*p* = 0.049).

A univariate Cox model showed that age and BCL7A expression were two survival related variables. A multivariate Cox model suggested that low BCL7A expression was an independent risk factor for ovarian cancer patients’ overall survival (HR = 1.52, *P* = 0.005, Table 3).

**Table 3:** Univariate and Multivariate Cox analysis of overall survival in ovarian cancer

Parameters	Univariate analysis			Multivariate analysis		
	Hazard.Ratio	CI95	*P*value	Hazard.Ratio	CI95	*P*value
age	1.63	1.19–2.24	0.003	1.64	1.2–2.26	0.002
subdivision	0.84	0.67–1.04	0.101			
stage	1.09	0.8–1.5	0.581			
longest dimension	1.12	0.82–1.52	0.485			
lymphatic invasion	1.02	0.85–1.23	0.798			
histologic grade	1.12	0.88–1.42	0.349			
sample type	0.43	0.11–1.73	0.235			
BCL7A	1.51	1.13–2.03	0.006	1.52	1.14–2.05	0.005

### The decrease in BCL7A expression is associated with poor relapse-free survival in ovarian cancer

**Fig. 3:**
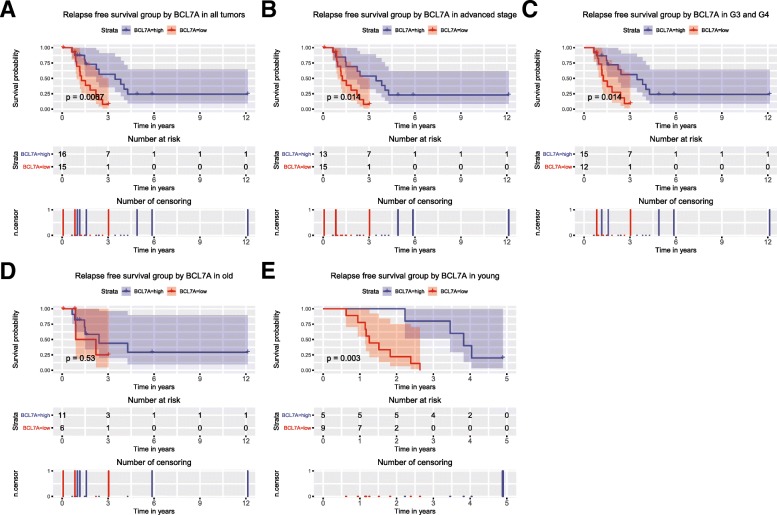
Survival analysis of BCL7A expression in terms of relapse-free survival. Kaplan–Meier curves produced survival analysis (**a**) and subgroup analyses of advanced stage (**b**), histological grade G3/G4 (**c**), older patients (**d**) and young patients (**e**)

As shown in Fig. 3, patients with a shorter relapse-free survival time had particularly low expression of BCL7A (*P* = 0.067), which was consistent with the results of subgroup analysis, especially in advanced stage (*P* = 0.014), G3 and G4 (*p* = 0.0014), and young patients (*p* = 0.003).

A univariate Cox model showed that BCL7A expression was the only relapse related variable. A multivariate Cox model suggested that low BCL7A expression was an independent risk factor for ovarian cancer patients’ relapse-free survival (HR = 3.65, *P* = 0.011, Table 4).

**Table 4:** Univariate and Multivariate Cox analysis of relapse-free survival in ovarian cancer

Parameters	Univariate analysis			Multivariate analysis		
	Hazard.Ratio	CI95	*P*value	Hazard.Ratio	CI95	Pvalue
age	0.71	0.3–1.67	0.433			
subdivision	0.92	0.34–2.48	0.875			
stage	1.92	0.78–4.71	0.156			
longest dimension	0.62	0.25–1.56	0.311			
lymphatic invasion	0	0-Inf	0.998			
histologic grade	1.35	0.65–2.83	0.421			
sample type	1.47	0.19–11.3	0.711			
BCL7A	3.65	1.35–9.89	0.011	3.65	1.35–9.89	0.011

## Discussion

Although the research on gynaecological cancer treatment technology is developing rapidly, predicting the outcome of ovarian cancer is still obscure [9]. So far, the treatment of ovarian cancer is mainly based on surgery and chemotherapy [10, 11]. Therefore, it is urgent to find novel biomarkers to predict the outcome of ovarian cancer, which can be used develop targeted therapy for ovarian cancer, especially in its early stages, to improve its survival rate and the patients’ quality of life. In this study, we found that the overall survival time of ovarian cancer patients was shorter when BCL7A was expressed at low levels. A Cox regression model showed that low expression of BCL7A was an independent risk factor for a poor outcome of ovarian cancer patients.

BCL7A was originally cloned from the chromosome translocation of the Burkitt lymphoma cell line [7] and it can interact with SWI/SNF components, suggesting that BCL7A participates in the progression of cancer cells by chromatin remodelling [12]. Recent studies have found that BCL7A expression is increased in diffuse large B-cell lymphoma (DLL) [13] but decreased in mycosis fungoides (MF) and peripheral T-cell lymphoma (PTCL) [14, 15]. Unlike these studies, we have found that BCL7A expression was downregulated in ovarian cancer tissues compared with normal ovarian tissues, which might be due to the different expression and functions of BCL7A in different tissues. In addition, the boxplots showed that the expression of BCL7A had no significant difference in regard to age, lymphatic invasion, stage, histologic grade, subdivision, new type, longest dimension, sample type, or vital status. Therefore, it is necessary to understand the role of BCL7A in ovarian cancer further.

BCL7A has been proven to have a close correlation with the prognosis of patients. Previous studies have found that early-stage cutaneous T-cell lymphoma patients with low expression of BCL7A were more likely to have a poor prognosis [8]. However, the association between BCL7A and overall survival was previously unknown in ovarian cancer. In this work, we found that the overall survival time was significantly shorter when ovarian cancer patients had low BCL7A expression. In addition, subgroup analysis found the same phenomenon in advanced stage, G3 and G4, and young patient and older patient groups.

To the best of our knowledge, this is the first study to examine the prognostic value of BCL7A expression in ovarian cancer. Together with other studies on the functions of BCL7A, we have contributed to a better understanding of the role of BCL7A as well as a better possibility of achieving a precise prognosis. However, the underlying mechanism has not been completely explored. In the future, we will perform sophisticated vitro and vivo experiments to explore the mechanism in depth.

## Conclusion

In conclusion, we generally focused on the prognostic value of BCL7A for ovarian cancer patients. Low BCL7A expression has been proven to be a distinct marker of patients with a poor prognosis. In the future, we plan to perform in vitro and in vivo experiments to explore the mechanism further.

## Materials and methods

### Data sources

We have obtained currently available clinical and RNAseq data from both normal and cancerous ovarian tissues. Databases, including TCGA (https://cancergenome.nih.gov/) and GTEx (www.gtexportal.org/), were utilized. Level 3 data were downloaded from the databases. This dataset shows the gene-level transcription estimates, as in log2(x + 1) transformed RSEM normalized counts. There is no existing ethical conflict because all data we used in this study are permitted to be used in research.

### Data mining

R (version 3.5.1) [16] was used for data mining. The ggplot2 package [17] was utilized to draw boxplots of clinical characteristics due to their expression variation. We used the median BCL7A expression as a cut-off to delineate the low vs high categories. The Chi-square test and Fisher’s exact test were used for the evaluation of the possible clinical correlation between clinical features and BCL7A expression. Survival package [18, 19] was used to draw survival curves, followed by log-rank tests to examine the survival deviation. Correlative variables were selected by a univariate Cox model, while the multivariate Cox model evaluated the independence of BCL7A rather than other clinical characteristics.
